# Wandering spleen; a rare clinical presentation of chronic pain with acute torsion

**DOI:** 10.1002/ccr3.8270

**Published:** 2023-11-27

**Authors:** Shahab Ghasemi, Dorsa Najari, Mohammadmoein Mirhosseini, Meisam Refaei

**Affiliations:** ^1^ Department of General & Vascular Surgery, Shohadaye Tajrish Hospital Shahid Beheshti University of Medical Sciences Tehran Iran

**Keywords:** case report, splenectomy, splenic torsion, wandering spleen

## Abstract

Wandering spleen results from abnormal ligamentous laxity and is often symptomatic, presented with abdominal pain and other non‐specific symptoms. These symptoms, make the diagnosis very difficult and most of the times even impossible. As such, keeping in mind this pathology, can make further complications less frequent.

## Graphical abstract

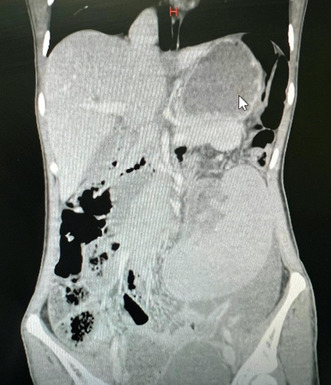

## 1 BACKGROUND

If a normal spleen is not identified in the left upper quadrant, a search for ectopic splenic tissue should ensue. If the patient has not had a prior surgical splenectomy, some possible explanations include an ectopic or “wandering” spleen, heterotaxy syndromes, splenosis, or sickle cell infarction/autosplenectomy.

Wandering spleen is a rare clinical entity with a less than 0.2% reporting incidence rate, which the spleen can be found in a number of positions in the abdomen or pelvis, and this condition is a result of congenital malformation or agenesis of the splenic ligaments or ligamentous laxity due to trauma, pregnancy, and connective tissue diseases.^1^ It can present with various clinical manifestations, ranging from asymptomatic to acute abdomen, which may require immediate surgical intervention. In this situation, radiological data is a great help and diagnostic imaging, particularly CT, allow radiologists to narrow the broad range of possibilities for a patient's nonspecific abdominal pain, and provide a precise diagnosis that would be nearly impossible without imaging.^2^ Although many conservative methods have been reported for the treatment of wandering spleen, the safest option is accepted to be surgery.^3^

Given the high incidence of life‐threatening complications, in the case of splenic torsion and infarction in these patients it is very important to promptly recognize this condition and initiate appropriate treatment. In this article, we report a young woman presented with abdominal pain to a remote hospital in Iran.

## 2 CASE REPORT

A 25‐year‐old woman complaining of acute abdominal pain, presented to the hospital with an onset date of 4 days ago in the left quadrant that could not be tolerated. Her pain started in the epigastric area and then radiated to the LLQ (Left Lower Quadrant) and LUQ (Left Upper Quadrant). It was positional, exacerbated in the supine position, and got better in the sitting position, but it did not radiate to any other area. She stated that she had this pain since a couple of years ago, when she had the history of an IUFD (Intra‐Uterine Fetal Demise) in the 29 weeks of pregnancy, but it had worsened a lot since 4 days ago. She also said that she could feel something moving in the exact region. She also complained of oligomenorrhea and headaches, which were followed by severe hypotension episodes and hospital visits. In her drug history, she only used h‐2 blockers. In her older documents, she had normal sonography. In the physical examination, she had tenderness in the LUQ and LLQ, and a vague giant lump was palpated in the LLQ, where the maximum tenderness was in that area, with no rebound tenderness or guarding.

Laboratory data did not show any notable pathology, other than an abnormal complete blood cell, which indicated iron deficiency anemia, leukocytosis and thrombocytopenia.

**FIGURE 1:**
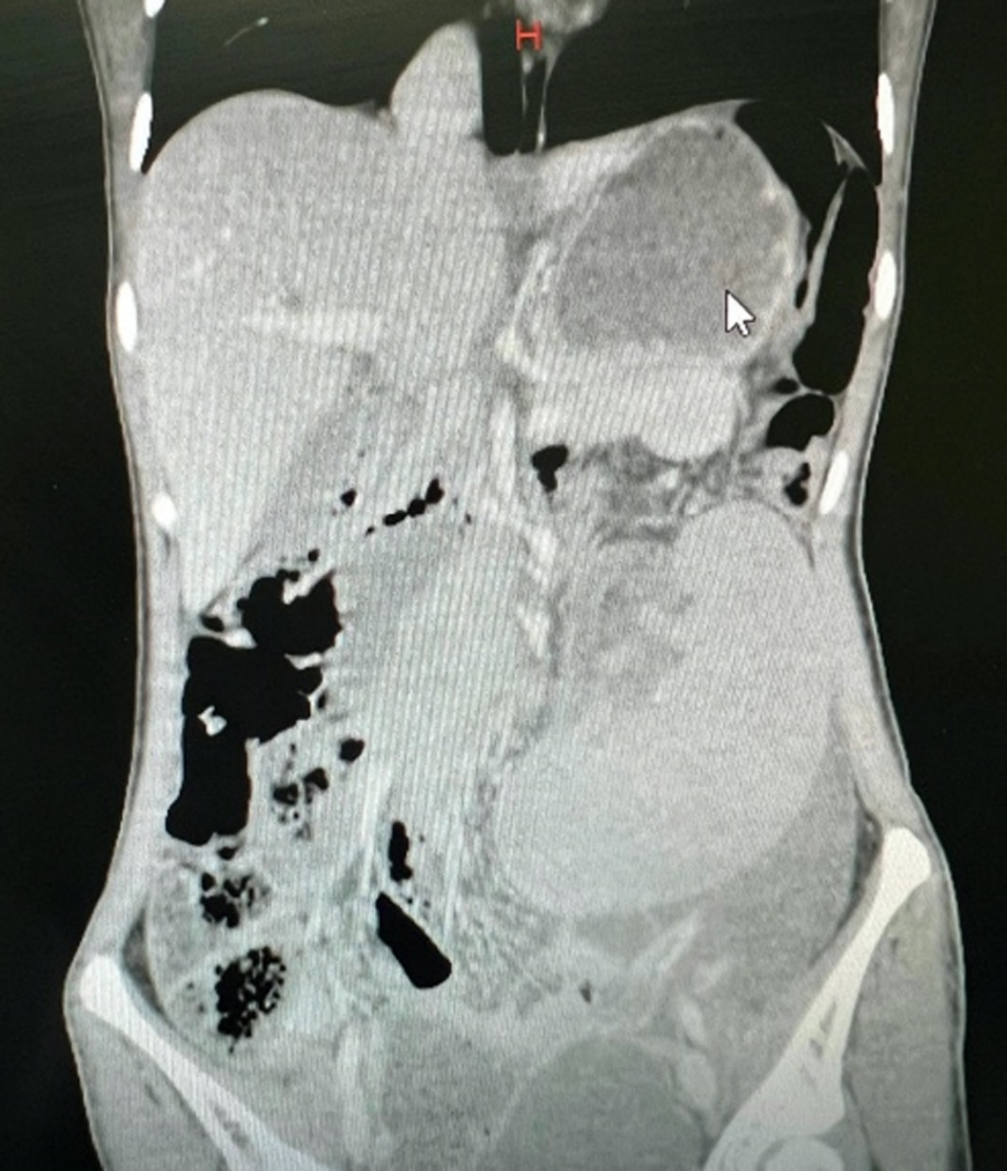
Computed tomography of patient, revealing a mispositioned spleen before surgery.

The abdominal X‐ray revealed an ileus without free air or air‐fluid levels in imaging modalities. An ultrasound revealed a low‐lying, 145 × 43 mm spleen that was absent from the left upper quadrant and the umbilical region of the abdomen. Torsion was not found. A CT scan was done to validate the findings. The diagnosis of a wandering spleen was confirmed by a CT scan, which also revealed splenomegaly and partial thrombosis in the splenic vein, as shown in Figure 1. We made the patient ready for surgery due to imaging modalities, leukocytosis, thrombocytopenia, and acute abdomen symptoms in the physical exam. A laparotomy was then conducted, which revealed a noticeably enlarged spleen that was not visible in its typical position. Exploration of the hilus region revealed a mild splenic vein thrombosis and ischemia as a result of a clockwise torsion. There, the stomach was also positioned incorrectly, and we had to gently realign it, but there was no suspicion regarding stomach volvulus, so no further actions were taken.

All of the aforementioned information led to the need for a splenectomy. Recovery after surgery went without incident. On the seventh day following the operation, she was released after receiving a polyvalent vaccination.

## 3 DISCUSSION

It seems that only <0.5% of splenectomies are due to ectopic positioning of the spleen, but because this condition is often asymptomatic and undetected, the exact incidence is unknown. However, a strong female predominance is described, with the average age of presentation of symptomatic patients ranging from 20 to 40 years of age.^4^ This may be due to the hormonal effect on the ligaments in multiparous women or injuries that cause the ligaments to weaken, such as connective tissue disease or pregnancy.^5^ In most of the cases discussed by our colleagues, patients were multiparous, that showed a rather important link with this matter. However, our patient was not multiparous, having only one alive birth and one IUFD.^1^, ^2^, ^3^, ^4^, ^5^, ^6^ Others seem to have a hematological underlying disorder, which make them more prone to spleen related diseases, but the patient discussed in our article did not have any previous medical history either, which show that it is important that we have to think of this diagnosis, even in least possible settings.^7^

Wandering spleen cases may be asymptomatic unless splenic torsion takes place. However, there may be an increase in inflammatory markers or signs and symptoms of hypersplenism or functional asplenia. Laboratory testing are typically unspecific. Abdominal pain, leukocytosis, nausea, vomiting, and peritoneal irritation are common symptoms of splenic torsion in patients. In worst‐case circumstances, compression of surrounding organs may result in gastric outlet obstruction, obstructive uropathy, duodenal obstruction, and portal hypertension.^6^ Our patient profile was in line with acute abdomen, however it seems that her pain was more chronic, which is not compatible with profile of mesentery acute torsion, symptomatic splenic infarcts, or splenic congestion, so we suspected that maybe her pathology was more an acute on chronic situation. Moreover, in a similar setting, an article by Ho described a 66‐year‐old Chinese woman with a past history of hemoglobin H (HbH) thalassemia, under the clinical impression of an ovarian mass. She complained of intermittent abdominal pain and distension, which persisted for 5 years and increased in frequency over the last 3 months, she was finally diagnosed with wandering spleen with chronic torsion.^8^

Due to the unspecific clinical features of abdominal pain, imaging modalities and radiologist's awareness of this condition play a crucial role in diagnosing ectopic spleen. These include ultrasonography, nuclear scintigraphy, contrast‐enhanced computed tomography (CT) scanning, magnetic resonance imaging and angiogram.^7^ Many studies, show that contrast‐enhanced CT is the best imaging tool to make this diagnosis. It is able to provide information about the exact location of wandering spleen in relation to other intra‐abdominal organs, and viability of the spleen in the setting of a possible splenic torsion, as well.^2^, ^3^, ^4^, ^5^, ^6^, ^7^, ^8^, ^9^

Surgical strategy changed over the time; splenectomy was the most reported treatment, but nowadays splenopexy is considered to be the optimal treatment for the non‐infarcted wandering spleen to avoid post‐splenectomy sepsis, however; many considerations have to be noted. A key consideration is the vascular status of spleen. Although imaging did not show us a problem, but during laparotomy evidence of ischemia and thrombosis was seen. Therefore, splenectomy was performed, but for a viable spleen, splenopexy is the treatment of choice.^1^, ^2^, ^3^, ^4^, ^5^, ^6^, ^7^, ^8^, ^9^, ^10^ Another point we have to keep in mind, is the age of patient. In children overwhelming post‐splenectomy infection is more common, so in our younger patient, saving the spleen is important.

## 4 CONCLUSION

Wandering spleen with torsion poses a great diagnostic challenge of acute abdomen in the emergency department due to the rarity of its occurrence. High index of suspicion, even in less probable patients is the key to early diagnosis and therefore splenectomy prevention. Due to this fact, splenopexy is considered to be the treatment of choice for these patients, if no other pathology is noted in the spleen.

## AUTHOR CONTRIBUTIONS

**Shahab Ghasemi:** Data curation; supervision; writing – review and editing. **Dorsa Najari:** Writing – original draft. **mohammadmoein mirhosseini:** Project administration. **Meisam Refaei:** Supervision; writing – review and editing.

## FUNDING INFORMATION

No funding sources.

## CONFLICT OF INTEREST STATEMENT

All authors have no conflict of interest to declare.

## ETHICS STATEMENT

Ethical approval was waived by the institution.

## CONSENT

Written informed consent was obtained from the patient to publish this report in accordance with the journal's patient consent policy.

## Data Availability

The datasets used and/or analyzed during the current study are available from the corresponding author upon reasonable request.

## References

[1] Virani P , Farbod A , Niknam S , Akhgari A . Wandering spleen with splenic torsion: report of two cases. Int J Surg Case Rep. 2021;78:274‐277. doi:10.1016/j.ijscr.2020.12.039 33373922 PMC7776122

[2] Reisner DC , Burgan CM . Wandering spleen: an overview. Curr Probl Diagn Radiol. 2018;47:68‐70. doi:10.1067/j.cpradiol.2017.02.007 28385371

[3] Termos S , Redha A , Zbibo R , et al. Torsion of huge wandering accessory spleen. Case report and review of literature. Int J Surg Case Rep. 2017;38:131‐135. doi:10.1016/j.ijscr.2017.07.037 28756363 PMC5537394

[4] Safioleas MC , Stamatakos MC , Diab AI , Safioleas PM . Wandering spleen with torsion of the pedicle. Saudi Med J. 2007;28(1):135‐136. 17206307

[5] Bhanumathi V , Balkishan B , Masood SV . Torsion of wandering spleen in a woman presenting as emergency. Indian J Surg. 2013;75(1):59‐61. doi:10.1007/s12262-012-0433-8 PMC3585543 24426389

[6] Cohen O , Baazov A , Samuk I , Schwarz M , Kravarusic D , Freud E . Emergencies in the treatment of wandering spleen. Isr Med Assoc J. 2018;20:354‐357. 29911755

[7] Vaynshtein J , Guetta O , Replyanski I , et al. Wandering spleen: three subsequent cases in young women. Isr Med Assoc J. 2018;20:656‐657. 30324789

[8] Ho CL . Wandering spleen with chronic torsion in a patient with thalassaemia. Singapore Med J. 2014;55(12):e198‐e200. doi:10.11622/smedj.2014185 25630326 PMC4292010

[9] Gayer G , Zissin R , Apter S , Atar E , Portnoy O , Itzchak Y . CT findings in congenital anomalies of the spleen. Br J Radiol. 2001;74:767‐772. doi:10.1259/bjr.74.884.740767 11511506

[10] Zarroug AE , Hashim Y , El‐Youssef M , et al. Wandering spleen as a cause of mesenteric and portal varices: a new etiology? J Pediatr Surg. 2013;48(3):e1‐e4. doi:10.1016/j.jpedsurg.2012.12.042 23480940

